# Supplementary material for: Crosstalk Between ALPK1 and STING: A Synergistic Axis in Innate Immune Activation and Human Inflammatory Disease
Source: bioRxiv. 2025 Jul 4:2025.06.30.662363. Preprint. [Version 1] doi: 10.1101/2025.06.30.662363 (PMC12236711; doi:10.1101/2025.06.30.662363)
Supplement: Supplement 1 [file media-1.pdf]

Supplement

Crosstalk Between ALPK1 and STING: A Synergistic Axis in Innate Immune Activation and Human Inflammatory Disease

Authors: Chong-Shan Shi, Christina Kozycki, Ning-Na Huang, Il-Young Hwang, Dima A. Hammoud, Daniel L. Kastner, and John H. Kehrl

Contents

Supplement fig. 1, parts 1-3.

Movie legends

Supplement movie 1-4.

Supplement fig. 1

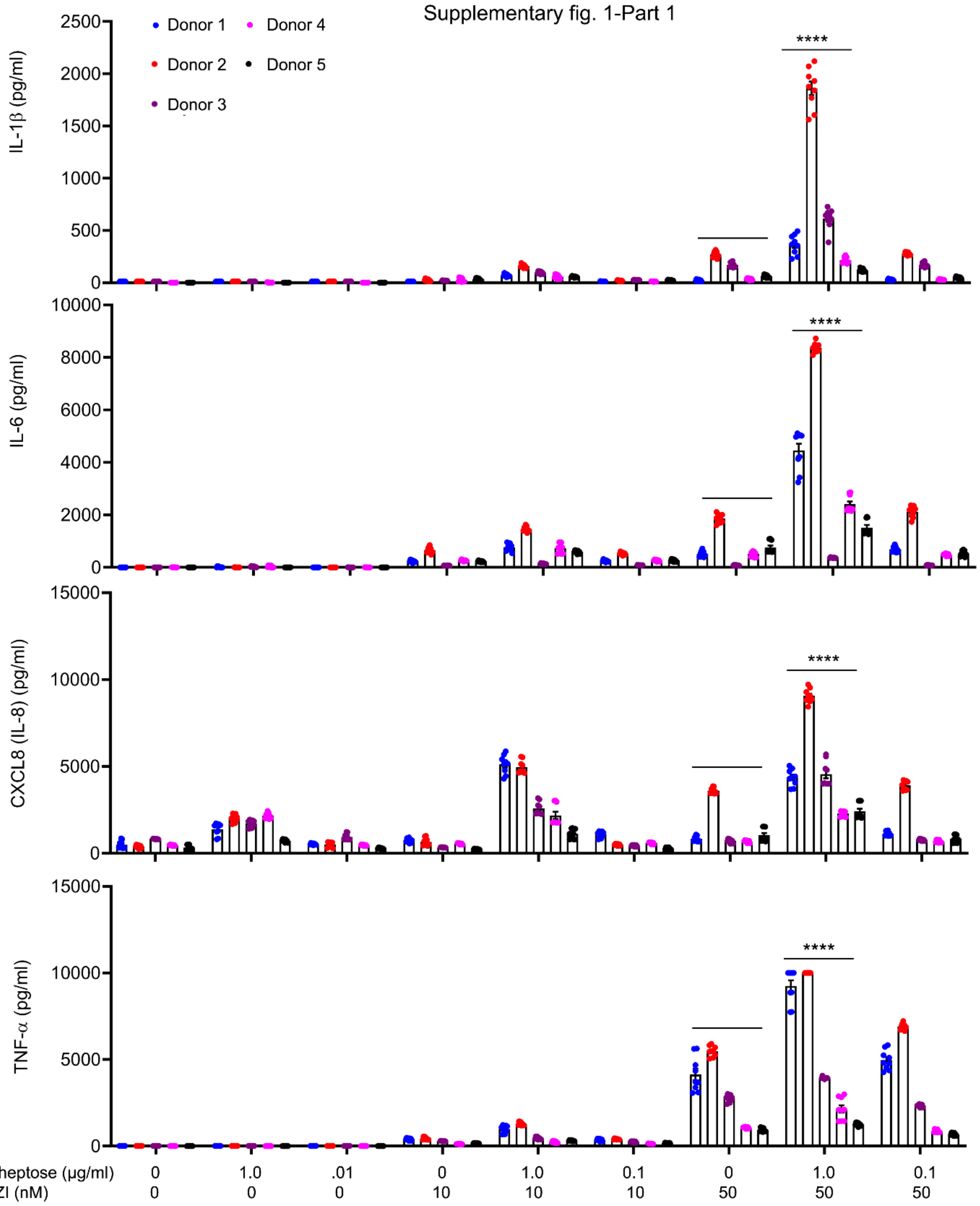

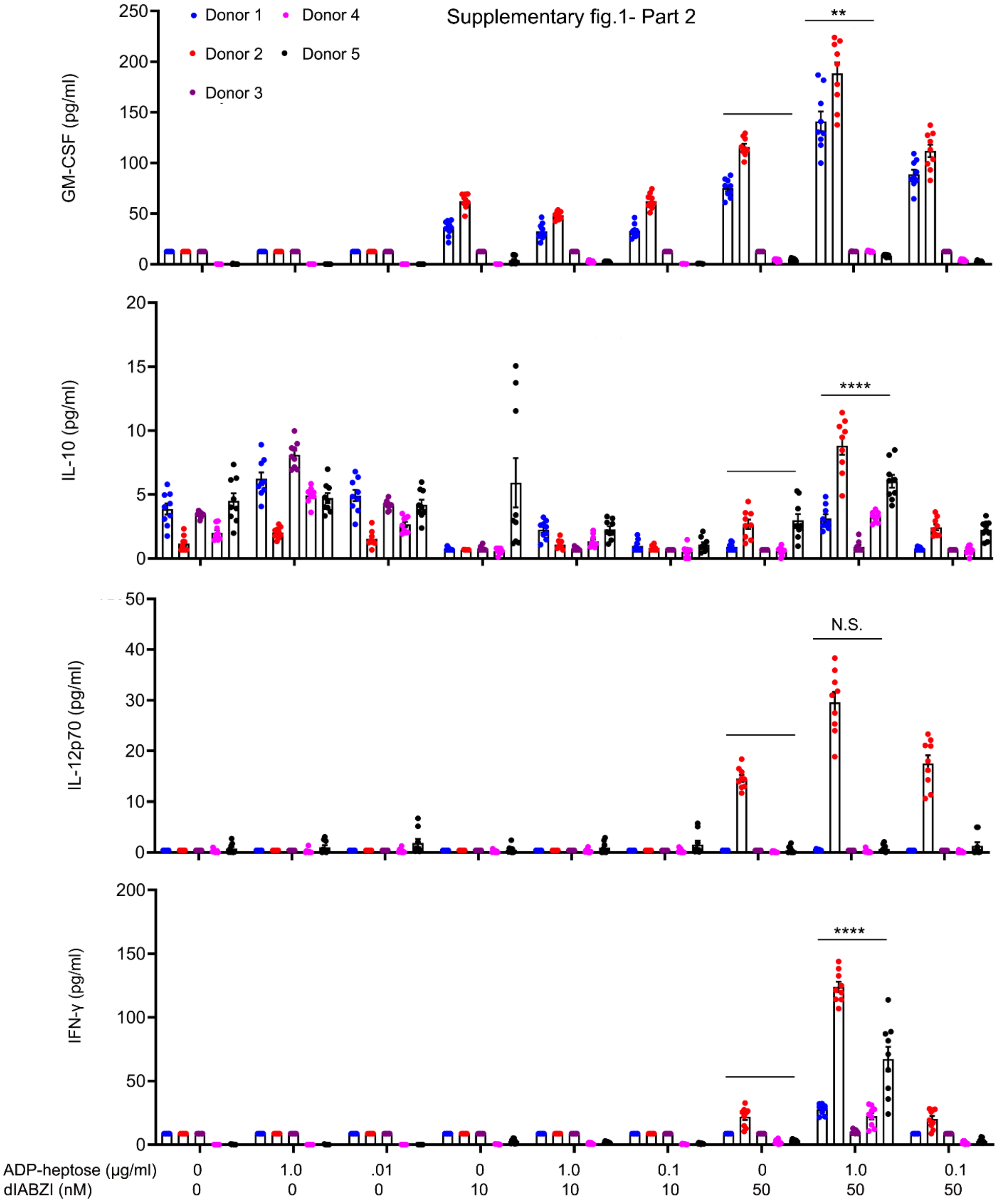
Supplementary fig.1- Part 2
Donor 1
Donor 2
Donor 3
Donor 4
Donor 5
GM-CSF (pg/ml)
IL-10 (pg/ml)
IL-12p70 (pg/ml)
IFN-γ (pg/ml)
**
****
N.S.
****
ADP-heptose (µg/ml) 0 1.0 .01 0 1.0 0.1 0 1.0 0.1
dIABZI (nM) 0 0 0 10 10 10 50 50 50

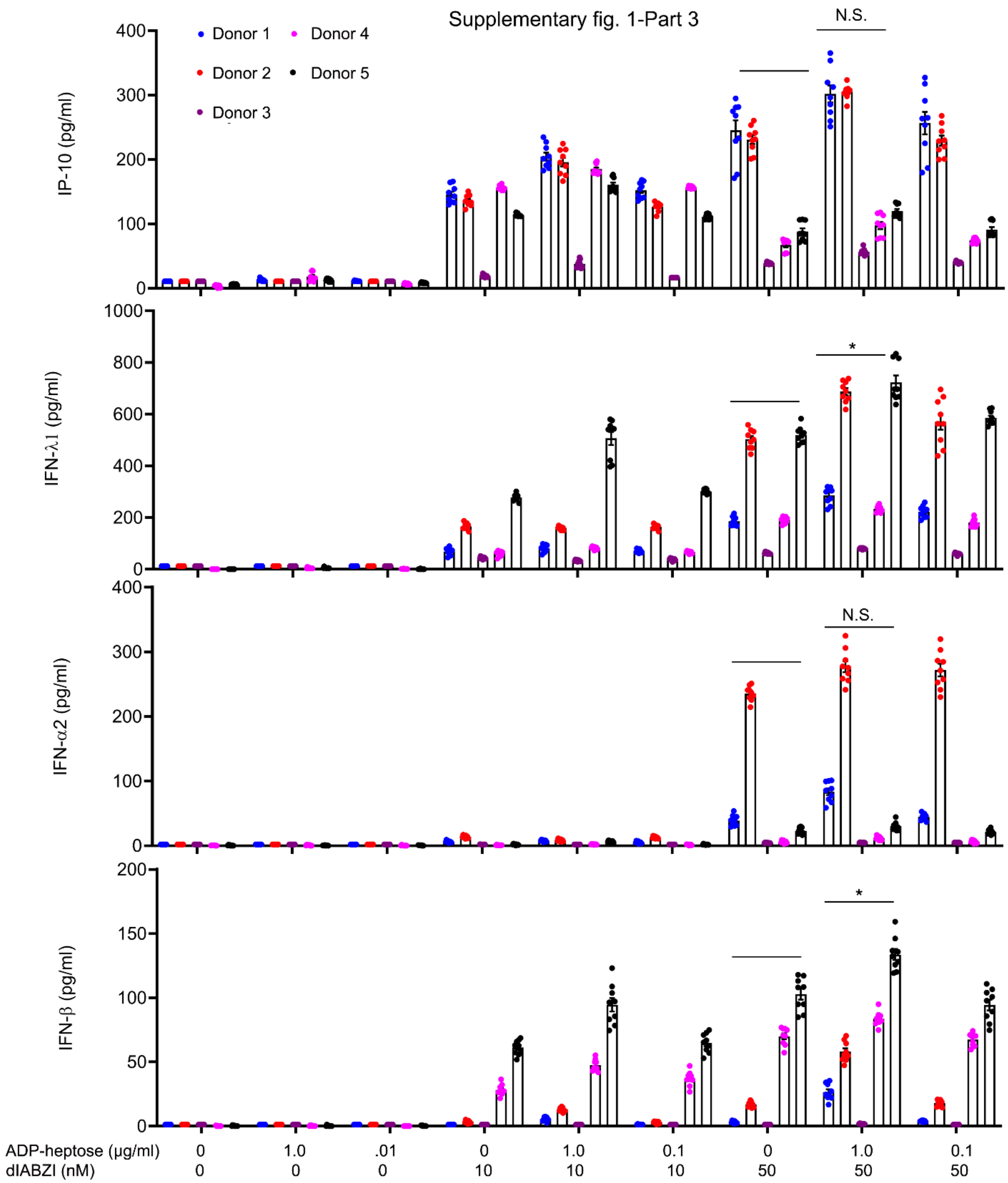

Supplementary fig. 1-Part 3
Donor 1
Donor 2
Donor 3
Donor 4
Donor 5
N.S.
*
N.S.
*
IP-10 (pg/ml)
IFN-λ1 (pg/ml)
IFN-α2 (pg/ml)
IFN-β (pg/ml)
0
100
200
300
400
1000
800
600
ADP-heptose (µg/ml) 0 1.0 .01 0 1.0 0.1 0 1.0 0.1
dIABZI (nM) 0 0 0 10 10 10 50 50 50

Supplementary fig. 1. Cytokine production by human monocytes from 5 donors.

Human monocytes purified from peripheral blood stimulated with ADP-heptose, diABZI, or both. Three cell supernatants from each donor each assayed in triplicate for indicated cytokines by multi-analyte flow cytometry. Statistics for comparison between diABZI (50 nM) and diABZI (50 nM) plus ADP-heptose (1.0 µg/ml) using data from all 5 donors.

Movie 1. ALPK1 and STING co-expression in HeLa cells. Live cell confocal imaging of HeLa cells transiently transfected with ALPK1-emerald (green) and STING-mRuby (pink) was performed. On day 2 following transfection the cells were imaged every 2 minutes for 2 hours. ADP-heptose 1.5 µg/ml was added at frame 10. Movie frame rate is 10 frames/second. Scale bar 10 microns. A single confocal image is shown for each frame.

Movie 2. ALPK1 Enhances STING expression in HeLa cells. Same live cell imaging as shown in movie 1, but the expression level of STING was mapped using the color table file Custom 1 in the Imaris program. The color gradient is shown in Figure 3. Imaging and frame rate the same as movie 1.

Movie 3. ALPK1 and STING Co-expression in HeLa cells. Live cell confocal imaging of HeLa cells transiently transfected with ALPK1-emerald (green) and STING-mRuby (pink) was performed. On day 2 following transfection the cells were imaged every minute for 90 minutes, movie spans the last 55 minutes. ADP-heptose 1.5 µg/ml was added just prior to initiating imaging. Movie frame rate is 10 frames/second. Scale bar 2 microns. A single confocal image is shown for each frame. Green-pink overlap is white.

Movie 4. ALPK Y254C and STING co-expressed in HEK293T cells. Live cell confocal imaging of HEK293T cells transfected 2 days previously with ALPK1 Y254C-Emerald and STING-mRuby and treated with diABZI (1 µM) at time point 18. Cells were imaged every minute for 90 minutes. Movie frame rate is 10 frames/second. A single confocal image is shown for each frame. Scale bar 4 microns. Green-pink overlap is white.
